# Polystyrene nanoplastics affect the human ubiquitin structure and ubiquitination in cells: a high-resolution study†

**DOI:** 10.1039/d2sc04434j

**Published:** 2022-11-11

**Authors:** M. della Valle, G. D'Abrosca, M. T. Gentile, L. Russo, C. Isernia, S. Di Gaetano, R. Avolio, R. Castaldo, M. Cocca, G. Gentile, G. Malgieri, M. E. Errico, R. Fattorusso

**Affiliations:** Department of Environmental, Biological and Pharmaceutical Science and Technology, University of Campania – Luigi Vanvitelli Via Vivaldi 43 81100 Caserta Italy roberto.fattorusso@unicampania.it; Institute of Biostructures and Bioimaging-CNR Via Mezzocannone 16 80134 Naples Italy; Institute for Polymers, Composites and Biomaterials – CNR Via Campi Flegrei, 34, 80078 Pozzuoli Naples Italy mariaemanuela.errico@ipcb.cnr.it

## Abstract

Humans are estimated to consume several grams per week of nanoplastics (NPs) through exposure to a variety of contamination sources. Nonetheless, the effects of these polymeric particles on living systems are still mostly unknown. Here, by means of CD, NMR and TEM analyses, we describe at an atomic resolution the interaction of ubiquitin with polystyrene NPs (PS-NPs), showing how a hard protein corona is formed. Moreover, we report that in human HeLa cells exposure to PS-NPs leads to a sensible reduction of ubiquitination. Our study overall indicates that PS-NPs cause significant structural effects on ubiquitin, thereby influencing one of the key metabolic processes at the base of cell viability.

## Abstract 2

A high-resolution study of ubiquitin–polystyrene nanoparticle (PS-NP) interaction is reported. We also show the effects of PS-NPs on ubiquitination in HeLa cells.

## Graphical abstract

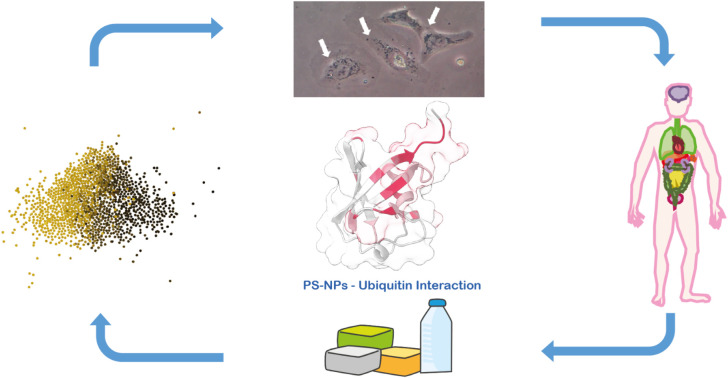

## Introduction

Among human-made materials, plastics are likely the most abundant; their increasing accumulation on the earth has been observed since 40 years and due to their chemical stability and they have been proposed as a stratigraphic marker for Anthropocene.^1,2^ More particularly, the world's plastic production approached 360 million tons in 2020 and it is estimated that by 2050, the production of plastic products will increase by 33 billions of tons.^3–5^ Despite all efforts to reuse and recycle end-of-life plastics, a large amount of plastic waste is leached into the environment, causing a severe pollution problem.^6^ Mechanical, photo-oxidative and biological processes induce the fragmentation of plastic materials, resulting in the formation of microplastics (MPs *d* < 5 mm) and nanoplastics (NPs *d* < 100 nm)^7^ which are distributed all over the planet. MPs and NPs have been detected, indeed, in soil, air, freshwater and oceans as well as in many foods, beverages and potable water.^8^ Through exposure to such a variety of contamination sources, humans are estimated to consume several grams of MPs and NPs every week.^2^ Nonetheless, these polymeric particles have unclear effects on organismal systems, in particular on humans. It has been suggested repeatedly that the exposure to NPs is likely higher and causes more severe problems than the exposure to MPs.^9,10^ It has been described that NPs can easily enter into cells and interact with various biomolecules due to their expansive surface to mass proportion and smaller size.^11^ Among biomolecules, proteins are of particular importance due to the high number of roles they play within living organisms. Changes in the structure can cause defects in these functions, which in some cases can result in the death of cells and organisms.^12^ Despite these considerations, only a few high-resolution experimental studies describing protein–nanoplastic interactions have been reported in the literature.^11–15^ Recently, the incubation of human plasma with nanoparticles obtained from daily-life polystyrene plastic items indicated the formation of a specific protein corona highlighting the needs for further studies to evaluate NP biological implications to determine their effects on biota.^15^ Ubiquitin, the partner of the proteasome in the ubiquitin–proteasome system (UPS), is a small protein of 76 amino acid residues (*M* = 8565 Da) folded into a compact globular structure in which a mixed parallel/anti-parallel β-sheet packs against an α-helix, generating a compact hydrophobic core.^16,17^ Ubiquitin plays a crucial role in cell proteostasis and ubiquitination is a powerful post-translational mechanism involved in a myriad of cellular signaling and proteasomal degradation pathways.^18,19^ Specific ubiquitination depends on the sequential cascade of substrate–ubiquitin chain building through an ATP-driven, a concerted process among three classes of enzymes: ubiquitin-activating enzymes (E1), ubiquitin-conjugating enzymes (E2), and ubiquitin ligating enzymes (E3).^20–24^

In this study, we have investigated, by means of CD, TEM and NMR methodologies, the interaction, at a molecular level, between polystyrene nanoparticles (PS-NPs) and ubiquitin. Particularly, we show that a hard protein corona is formed upon ubiquitin structural rearrangement, induced by PS-NPs, which allows ubiquitin self-interaction. Furthermore, we have monitored the effects of the PS-NPs on HeLa cells and particularly the levels of ubiquitination in these cells when they are in the presence of PS-NPs. Our data clearly outline the molecular details of the PS-NP interaction and show that PS-NPs may sensibly reduce ubiquitination in cell.

## Experimental section

### Polystyrene nanoparticles and ubiquitin samples

Polystyrene (PS) nano-particle 1% solids (Catalog #3020A) (NPs) were purchased from Thermo Fisher Scientific – Particle Technology, CA, USA.

^15^N–^13^C labeled human ubiquitin (Ubq), 10 mg 1 mM/50 mM phosphate buffer pH 7, 10% ^2^H_2_O, and 0.02% NaN_3_ were purchased from Giotto Biotech. S.r.l., IT.

### Circular dichroism (CD) measurements

CD spectra were collected in the 200–260 nm wavelength range on a JASCO J-815 CD spectropolarimeter equipped with a Peltier temperature controller using a quartz cuvette of 1 cm path length, with a data pitch of 1 nm, scanning speed of 50 nm min^−1^ and a bandwidth of 1 nm. CD samples were prepared by dissolving Ubq (25 μM) in 50 mM phosphate buffer at pH 7, and then titrating it with PS-NPs up to a maximum concentration of 0.15 mg mL^−1^. Each protein spectrum has been subtracted of the blank contribution; the buffer and the appropriate amount of NPs for each sample constituted the blank. The CD spectra of the NPs alone are reported in Fig. S1.†

### Nuclear magnetic resonance (NMR) spectroscopy

All NMR experiments were performed at 298 K by using a Bruker AVANCE III HD 600 MHz spectrometer equipped with a triple resonance Prodigy N_2_ cryoprobe having a *z*-axis pulse field gradient. NMR samples were prepared by dissolving ^15^N–^13^C labelled protein in 200 μL of 50 mM phosphate buffer, pH 7, and 10% ^2^H_2_O to reach a final Ubq concentration of 100 μM.

A standard set of triple-resonance NMR experiments was performed as previously reported to enable the assignment of the sequence-specific backbone and Cβ resonances;^25^^1^H–^13^C HSQC was used to assign H_α_s and H_β_s. NMR spectra were processed by using TopSpin 4.1.0 (Bruker) and analyzed using SPARKY^26^ and CARA software.^27^ Protein structures were visualized and evaluated using CHIMERA software.^28^

Ubq structural rearrangements induced by nano-polystyrene binding were estimated by applying combined ^1^H (Δ*δ*_H_), ^15^N (Δ*δ*_N_) and ^13^C (Δ*δ*_C_) Chemical Shift Perturbations (CSPs) based on the equations reported below:Δ*δ*_H_N_/N_ = ((Δ*δ*_H_*W*_H_)^2^ + (Δ*δ*_N_*W*_N_)^2^)^1/2^Δ*δ*_H_α–β_/C_α–β__ = ((Δ*δ*_H_*W*_H_)^2^ + (Δ*δ*_C_*W*_C_)^2^)^1/2^in which *W*_H_, *W*_N_ and *W*_C_ are the weighing factors for ^1^H, ^15^N and ^13^C shifts defined as *W*_H_ = |*γ*_H_/*γ*_H_| = 1; *W*_N_ = |*γ*_N_/*γ*_H_| = 0.101 and *W*_C_ = |*γ*_C_/*γ*_H_| = 0.251. Δ*δ*_H_, Δ*δ*_N_ and Δ*δ*_C_ are the chemical shift differences in ppm for ^1^H, ^15^N and ^13^C, respectively; *γ*_H_, *γ*_N_ and *γ*_C_ are the gyromagnetic ratios of the different nuclei.^29^ Ubq backbone dynamics upon nano-polystyrene interaction were explored by ^1^H–^15^N intensity profile analysis. The intensity variations were calculated by using the equation Δ*I* = *I*_bound_/*I*_free_, in which *I*_bound_ and *I*_free_ are the peak intensities in the presence and in the absence of PS-NPs, respectively.

#### H–D exchange

Lyophilized ^15^N–^13^C Ubq was resuspended in 200 μL of ^2^H_2_O in the presence of 0.6 mg mL^−1^ of PS-NPs to a final concentration of 300 μM. A series of 2D ^1^H–^15^N HSQC spectra were immediately collected at regular time intervals of 20 minutes up to 360 minutes. For real-time HDX measurements, the time-dependent intensity behavior of each atom was fitted by an exponential decay to obtain the *k*_ex_. The protection factor was calculated as *k*_intrinsic_/*k*_ex_, where the intrinsic exchange rates were calculated using the SPHERE server (https://protocol.fccc.edu/research/labs/roder/sphere/sphere.html).^30^

### Microscopic examination

Physical changes in the NPs that interacted with Ubq have been analyzed by using Transmission Electron Microscopy (TEM), using a FEI Tecnai G12 Spirit Twin with LaB_6_ source (FEI, Eindhoven, The Netherlands). Before the analysis, PS-NPs were diluted at 0.1 wt% content by adding distilled water and sonicated using a Sonics Vibracell (Newtown, CT, USA) ultrasonic processor (500 W, 20 kHz) at an amplitude of 25% for 10 min to promote the NP dispersion. Ubq was then added to the dispersion (Ubq : PS-NP weight ratio 1 : 1) and, and after 24 h of incubation, the Ubq–PS-NP sample was collected on carbon coated copper grids and water was let to evaporate at room temperature. TEM images were finally collected at 120 kV acceleration voltage in bright field mode using a FEI Eagle 4k CCD camera.

### In-cell studies

#### Cell culture

HeLa cells were cultured in Dulbecco's modified Eagle's medium (DMEM) supplemented with 10% fetal bovine serum and incubated at 37 °C with 5% CO_2_. HeLa cells were plated into a 6 mm Petri dish and, and the day after were treated with 0.2 ng mL^−1^, 0.2 μg mL^−1^ and 0.2 mg mL^−1^ of nanoplastics and incubated at 37 °C for 24 hours. Following incubation, each well was imaged directly under an inverted phase contrast microscope (Eclipse TE300, Nikon) at 10× magnification.

Cell viability was evaluated using the trypan blue exclusion test and MTT assay. In particular, after image acquisition, cells were detached from the dish with 0.25% trypsin, harvested by centrifugation at 1500 rpm for 3 min and resuspended in 0.5 mL of PBS. 0.2 mL of cell suspension were added to 0.5 mL of PBS and 0.3 mL of 0.4% of trypan blue solution (Lonza). After 5 min at room temperature, cells were counted in a Burker chamber. For each of the conditions tested, we also performed MTT assay to evaluate cell metabolism. In particular, cells were seeded at 3 × 10^5^ cells per well in a 24 well plate in the presence or absence of increasing doses of PS-NPs as indicated. Cell viability was assessed after 24 h. According to manufacturer's recommendations, 50 μL of 3-(4,5-dimethylthiazol-2-il)-2,5-diphenyl-2*H*-tetrazolium bromide (MTT; Sigma Aldrich, USA) reagent (5 mg mL^−1^ in PBS) was added to each well and then the cells were incubated at 37 °C for three hours. One volume (500 μL) of stop mix solution (20% SDS in 50% dimethyl formamide) was added to each well and incubated at room temperature for a minimum of 1 h. The plate was read at 550 nm and at 630 nm as the reference wavelength. The same volume of medium without cells was used as the blank. The results are expressed as OD.

#### Protein analysis

The isolation of cytosolic protein was performed by scraping cells in lysis buffer (25 mm Tris–HCl, pH 7.4, 1 mm EDTA, 1 mm EGTA, 0.1 mm NaF, and 0.1 mm Na_3_VO_4_). The protein concentration was determined by the Bradford method using the Bio-Rad reagent (Bio-Rad Laboratories, Hercules, CA, USA). 20 μg of protein extract were subjected to 10% SDS-PAGE and electroblotted on PVDF membranes, which were blocked at room temperature for 1 h in blocking solution (5% BSA in TBS-T), and incubated overnight at 4 °C with anti-ubiquitin (1 : 1000; Abcam). The membrane was incubated with peroxidase-conjugated anti-mouse antibodies (1 : 10 000; Amersham Biosciences). Proteins were revealed by using an ECL kit (Millipore) after 10 s exposure. Normalization was performed with anti-tubulin (1 : 1000 in TBS-T; Cell Signaling) for 1 h at room temperature and incubated with peroxidase-conjugated anti-mouse IgG (1 : 10 000 in TBS-T). Proteins were revealed as above. Band intensity was quantified by using Image Lab Software ChemiDoc (BioRad).

## Results and discussion

### Ubq/nano-polystyrene interaction at low resolution

**Fig. 1:**
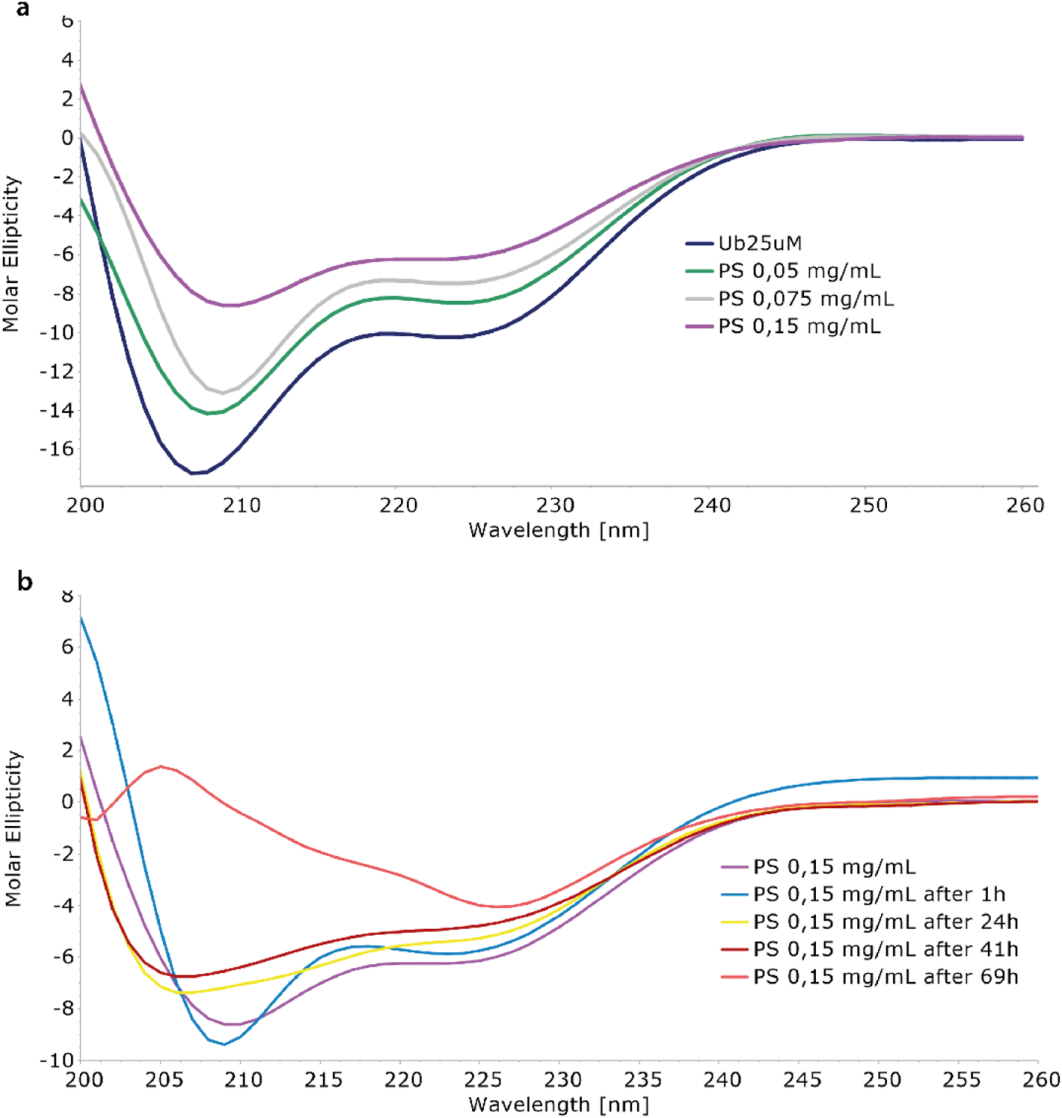
Effects of Ubq–NP interaction *via* CD. (a) CD titration of ubiquitin with different amounts of PS-NPs (figure legend reports the PS concentration added to the Ubq sample). (b) Time-depending changes in the Ubq CD spectra in the presence of PS-NPs.

The interaction between Ubq and PS-NPs has been investigated initially monitoring the protein structure in the presence of increasing amounts of PS-NPs by means of CD spectroscopy. The inspection of the CD spectra recorded in absence and in the presence of PS-NPs (Fig. 1a) confirmed the interaction of ubiquitin with PS-NPs and documented a conformational change in the Ubq structure upon interaction.^31^ The spectrum of Ubq alone was typical for a protein with both α-helix and β-sheet structures (Fig. 1a). The addition of PS-NPs led to a progressive change in the protein secondary structure,^12^ as indicated by the gradual increase in ellipticity along with spectral shape changes. Samples have been then incubated and monitored at regular intervals (Fig. 1b) showing an additional slight increase in the ellipticity and spectral shape changes also over time. These data indicate a destabilization of ubiquitin helices due to PS-NP binding compared to the free ubiquitin.^32^ The shift in the deep minimum near 210 nm of the initial spectra towards 205 nm is typical of an increase of unfolded protein.

After 1 hour of incubation, the α-helix band to some extent further decreases while the β-sheet band increases. The partial loss of the secondary structure^33^ is consistent with the formation of a hard corona of Ubq proteins surrounding NPs,^11,14^ a layer of tightly bound proteins with a long lifetime. In a soft corona, the external layer of “softly” bound proteins with a shorter lifetime and significant differences in the protein secondary content compared to the native state are not expected.^11^ The incubation of the sample led to dramatic changes in the CD spectra as a result of a reduction of protein in solution, confirming the activation of aggregation processes.^34^ At 69 hours, the data are quite atypical for protein CD spectra. The behavior here observed for Ubq is in line with the behavior observed in the case of human serum albumin, in which a reduction of the α-helical content was observed upon interaction with polystyrene NPs.^14,34^

### Protein-corona formation on nano-polystyrene

**Fig. 2:**
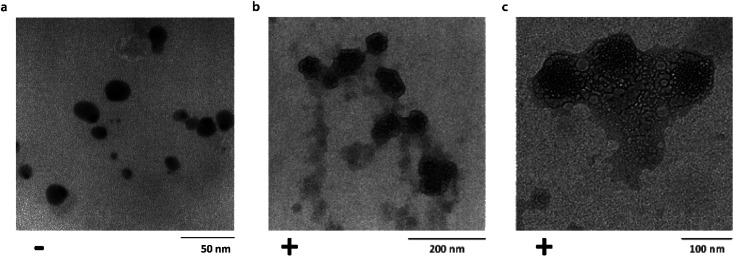
Corona formation. Untreated PS-NPs (diameter: 15 ± 4 nm) (a). Bright field TEM images of Ubq–NP after 24 h of incubation at (b) 200 nm and (c) 100 nm.

Transmission electron microscopy (TEM) was used to investigate single particle and aggregate morphologies in the Ubq–NP samples. TEM confirmed CD results concerning the binding of ubiquitin to PS-NPs (Fig. 2a–c). In particular, TEM images acquired after an incubation of 24 h at different magnifications show that, upon interaction, a thick layer of Ubq composed of several Ubq molecules surrounds spherical PS-NPs characterized by high electron-contrast (average diameter 54 ± 7 nm), stabilizing a hard corona that almost uniformly binds NPs. A corona is generally defined as a thin protein layer of a few nanometers, corresponding in some cases to a protein monolayer.^15,35^ Interestingly, the 24 h incubation period of the sample led in our case to a considerable grain growth. Moreover, it is possible to observe that, as expected, the protein induces coalescence of several coronated-NPs to produce large particle aggregates with a size up to about 300 nm.^11,12,34^

Such a protein corona embeds plastic nanoparticles forming a new complex structural entity; as a consequence, the protein functions may be altered affecting the cell metabolism.^36^

The hard protein corona offers a new character to PS-NPs^36,37^ determining its capability to interact and affect cells.^37^ For this reason, a thorough understanding of this particle–corona complex is essential to understand its role in NP toxicology. Moreover, NP interaction with proteins can alter protein conformation, expose different epitopes on its surface and perturb its normal function, inducing unwanted biological reactions. We thus resorted to deepen our study in two directions: performing detailed characterization at an atomic resolution level of the interaction between Ubq and PS-NPs by NMR and characterizing the impact of NPs on ubiquitination at a cellular level.

### Ubq/nano-polystyrene interactions at atomic resolution

**Fig. 3:**
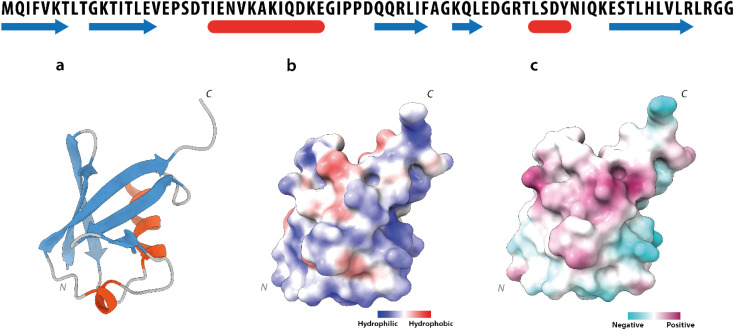
Ubiquitin structure. Upper part: ubiquitin amino-acid sequence. The secondary structure elements are depicted in blue (β-strands) and in red (α-helices). Lower part: (a) ribbon drawing of the ubiquitin X-ray structure (PDB code 1UBQ). (b) Solvent accessibility and (c) charge distribution of the ubiquitin surface.

Human Ubq is a 76 amino acid polypeptide that folds into a highly stable globular protein. It shows a prevalently polar surface and a solvent-exposed hydrophobic region, centered around residues leucine 8, isoleucine 44 and valine 70 ^17^ (Fig. 3a–c).

**Fig. 4:**
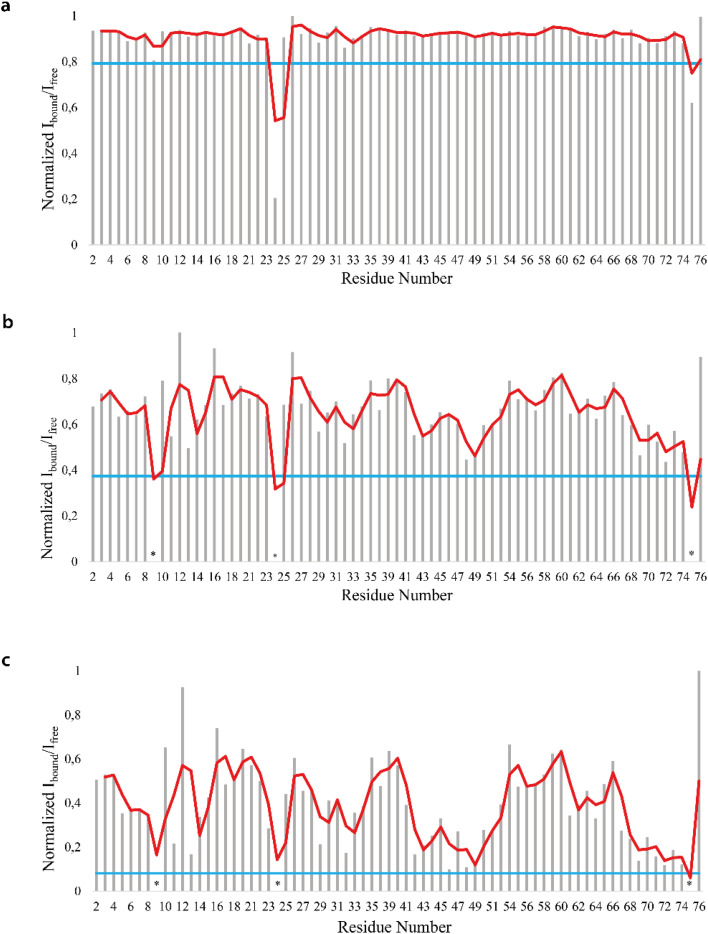
NMR signal intensity profile analysis. Normalized profile of the intensity variation of the H_N_–N correlation peaks in the Ubq ^1^H–^15^N-HSQC spectra in the presence of 0.1 (a), 0.2 (b) and 0.3 (c) mg mL^−1^ of PS-NPs. The blue line represents the mean + SD value.

**Fig. 5:**
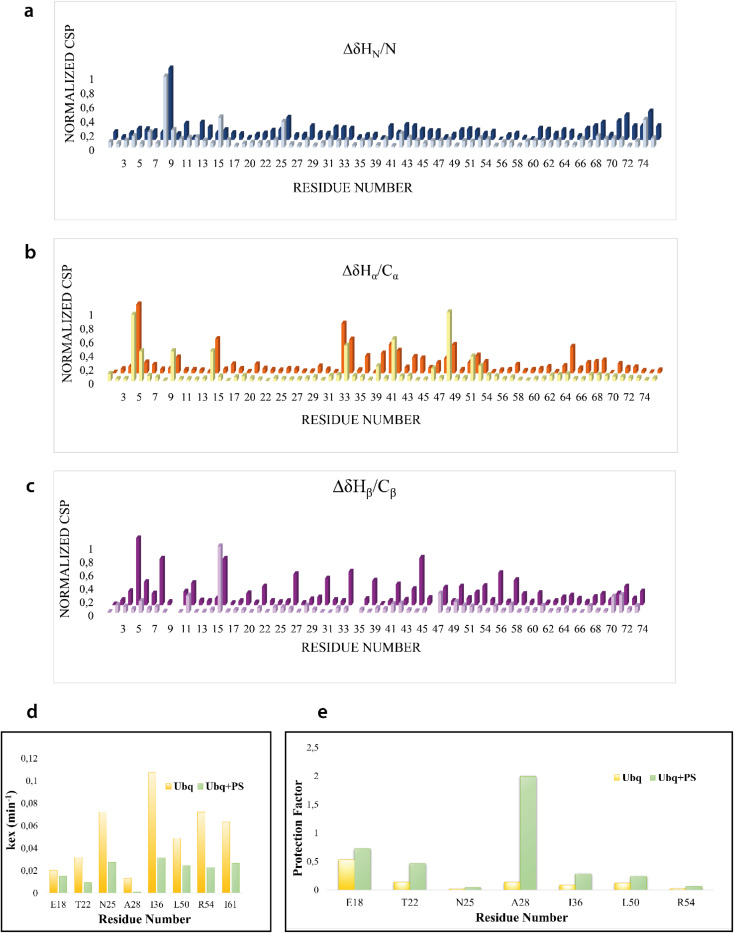
Chemical shift perturbation and hydrogen–deuterium exchange. Normalized CSP of Ubq H_N_/N (a), H_α_/C_α_ (b) and H_β_/C_β_ (c) after the addiction of 0.2 and 0.3 mg mL^−1^ of PS-NPs. Selection of *K*_ex_ (d) and protection factors (e) of Ubq free in solution (yellow) and in the presence of 0.6 mg mL^−1^ of PS-NPs.

We firstly characterized the free ^15^N–^13^C labeled Ubq by means of double and triple resonance experiments that constituted the reference spectra. The BMRB chemical shifts of ubiquitin (BMRB code 6457) were used to completely assign the recorded NMR experiments showing that the protein under the chosen conditions is monomeric and properly folded. The ubiquitin was then titrated with different aliquots (ng mL^−1^) of an aqueous suspension of polystyrene nanoparticles to investigate different Ubq/NP ratios (Fig. S2†). For every point a new set of bi and multinuclear NMR spectra was recorded. Ubq spectra, as expected, are perturbed by the interaction of the protein with NPs.^32^ The most evident effect is the broadening of protein signals (some even disappear) upon absorption of Ubq on NPs, due to the slower tumbling rate of the larger assembly.^16,38^ Ubq interaction with NPs gives rise to a dynamic association equilibrium, in which the perturbations of the observable signals are determined by the chemical exchange regime.^16^ The signals of Ubq bound to NPs are, as expected, invisible due to line-broadening but information regarding the bound state can be obtained due to the exchange-mediated signal averaging.^16,39,40^ Chemical shift perturbation and signal intensities with respect to reference spectra as a function of ligand addition thus provide useful dynamic and conformational information about the binding event. For this reason, the chemical shifts and intensity variations of the different nuclei in the presence and absence of NPs were evaluated. The analysis of Ubq HSQC after NP addition shows that the overall fold of the protein is maintained throughout the titration. A certain number of resonances of many amino acids were perturbed, broadened and/or shifted indicating both the direct involvement of these amino acid residues in the binding event and their proximity to the binding surface. Plots showing the perturbation of signal intensities and backbone chemical shifts are reported in Fig. 4 and 5, respectively. As shown in Fig. 4a–c, the signal intensity profile starts to be slightly perturbed after the addiction of 0.1 mg mL^−1^ of NPs. The affected residues, *i.e.* threonine 9, glutamate 24 and glycine 75, belong to the N-terminus of the α-helix, the β1–β2 turn and the C-terminus of the protein respectively; the rest of the structure does not show any significant intensity variation. The broadening of these signals is accompanied by the chemical shift perturbation of the residues surrounding them (Fig. 5, panels a–c), indicating a local rearrangement of the protein structure.^33^ It is important here to note that the C-terminal part of the ubiquitin is responsible of the E1–Ubq conjugation, a crucial step of the ubiquitination process.

**Fig. 6:**
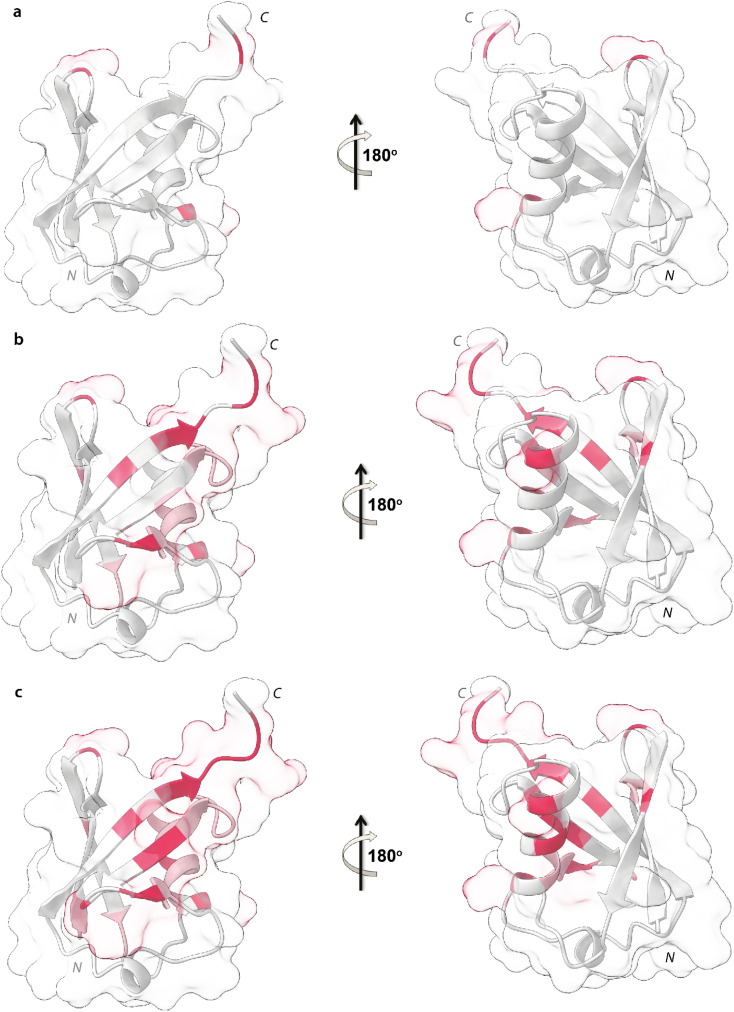
Mapping of the intensity profile analysis. Mapping of the Ubq structure of the most affected residues in the presence of 0.1 (a), 0.2 (b) and 0.3 (c) mg mL^−1^ of PS-NPs.

Upon addition of 0.2 mg mL^−1^ and 0.3 mg mL^−1^ of NPs, most of the signals of Ubq further decrease in intensity. These residues reported in Fig. 6 (panel a–c) and labeled on the protein structure give a clear indication of the protein portions involved in the interaction with NPs.

Ubq interacts with NPs using a region that encompasses the C-terminal tail and the large surface constituted by the last three strands of the β-sheet. This statement finds support in the nature of the perturbed amino acids (Fig. 3c) capable of driving the electrostatic adsorption of Ubq on polystyrene NPs.^32^ At 0.2 mg mL^−1^ and 0.3 mg mL^−1^ of NPs, also the chemical shift perturbations become more evident. Δ*δ* analysis of the nuclei of the backbone (HN/N, Hα/Cα and Hβ/Cβ) shows global differences in CS for all the backbone atoms, suggesting a reorganization of the protein structure in clear agreement with the CD data. Interestingly, also isoleucine 13 in the first β-strand along with lysine 29 and aspartate 32 of the first helix is clearly broadened upon NP addition defining a second region influenced by the binding event. This second region, however, is located on the opposite face of the protein with respect to the region that we ascribe to directly interact with NPs. This behavior, also in light of the TEM analysis, finds an explanation in the fact that the global rearrangement of the protein structure allows the formation of protein–protein interactions that bridge different Ubq monomers, which constitute the secondary binders, associated with the primary binders (Ubq directly bound on NPs) through protein–protein interactions and allows the coalescence of aggregates.^34^

H–D exchange experiments (Fig. S3†) were also performed in the presence of (0.6 mg mL^−1^) NPs but the complex equilibrium in solution, here descripted, did not allow the evaluation of the amide protection factors from solvent exchange for most of the protein residues; Fig. 5d and e, however, report the *K*_ex_ and the protection factors for those residues that we were able to properly evaluate and their comparison to the same residues of the protein free in solution. They support significant differences in the properties of the two proteins.^41,42^ A general increase in protection factors is clearly evident in the presence of NPs. In particular, the residues reported in the figure all belong to flexible regions linking the secondary structure elements. These regions, as such, are endowed with a conformational exchange that in the free protein results in a faster exchange with the solvent with respect to those residues located in secondary structure elements. The presence of NPs sensibly slows this exchange indicating a general change in the internal dynamic properties of the protein and again suggests a global structural rearrangement of the protein structure consistent with the CD and NMR data here reported. Ubq structural rearrangements upon nanoparticle binding are in perfect agreement with the study reported by Ding *et al.*^32^ in which the authors show that Ubq binding to AgNPs (silver nanoparticles) led to a slight change in the secondary structure content of the protein.

Summarizing, the here reported *in vitro* data demonstrate that the interaction of NPs with Ubq overall results in three important phenomena: the formation of a protein corona, the coalescence of NPs driven by the protein and changes in the Ubq structure.

### Ubq/nano-polystyrene interactions at the cellular lever

**Fig. 7:**
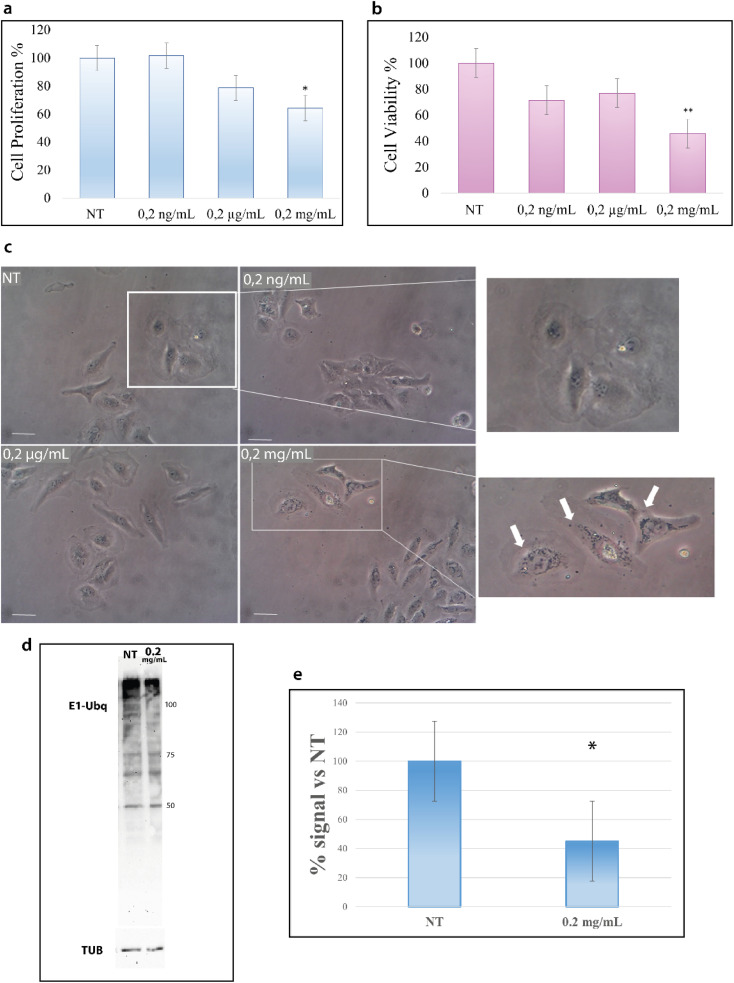
PS-NPs interfere with eukaryotic cell metabolism, localize into the cell cytoplasm and inhibit the ubiquitination reaction. (a) Proliferation assay on HeLa cells treated with increasing doses of PS-NPs for 24 hours (trypan blue exclusion test); * <0.01 *versus* NT. (b) Metabolic assay on HeLa cells treated with increasing doses of PS-NPs for 24 hours (MTT assay); ** <0.001 *versus* NT. (c) Representative high-power images of HeLa cells treated with increasing doses of PS-NPs for 24 hours. Images were captured from untreated cells (NT) and cells treated with 0.2 ng mL^−1^, 0,2 μg mL^−1^ and 0.2 mg mL^−1^ of PS-NPs with 10× magnification. Scale bar is 250 μm. (d) Representative western blotting analysis of the Ubq signal in HeLa cells untreated (NT) and treated with 0.2 mg mL^−1^ for 24 hours. The image is representative of four independent experiments. (e) The graph represents the relative densitometric analysis of the signal intensity of the four experiments; data are represented as a ratio of Ubq normalized *versus* tubulin and indicated as % of control conditions (samples derived from the same experiment are considered); * *p* < 0.05 *vs.* control conditions. All the experiments have been performed in parallel. Proteins were revealed by using an ECL kit (Millipore) after 10 s exposure, images were obtained by using Image Lab ChemiDoc and no manipulations had been performed.

HeLa cells were treated with increasing concentrations of NPs; particularly, cells were incubated for 24 hours at 37 °C with 0.2 ng mL^−1^, 0.2 μg mL^−1^ and 0.2 mg mL^−1^ of NPs and then analyzed with the trypan blue exclusion test for cell viability and with MTT assay for metabolism. As shown in Fig. 7a, the treatment with NPs results in a decrease of cell proliferation that starts at 0.2 μg mL^−1^ and becomes considerable at 0.2 mg mL^−1^.

Concurrently, we evaluated the cytotoxic effect of NPs by MTT assay. MTT assay measures the activity of NADH/NADPH oxidase in the endoplasmic reticulum and the activity of succinate dehydrogenase in mitochondria, two important steps in cellular metabolism. As shown in Fig. 7b, the treatment with NPs induces a reduction in cell metabolism^13^ that becomes particularly significant at 0.2 mg mL^−1^.

The results of these two assays suggest the interference of NPs with cell metabolic machinery and prompted us to evaluate whether NPs are able to be internalized into the cytoplasm.^43^ The images in Fig. 7c show the accumulation of black particles in the perinuclear region (as indicated by white arrows) of cells treated with NPs,^44^ particles that are not visible in untreated cells, suggesting that NPs can enter the cells and accumulate into the cytoplasm.^15^ In agreement with the data of the cell viability tests, NP internalization starts to become evident at 0.2 mg mL^−1^.

Such results encouraged us to investigate also in a cellular context the influence of NPs on the ubiquitination reaction. Cells were thus treated with 0.2 μg mL^−1^ and 0.2 mg mL^−1^ NPs for 24 hours, as for the previous experiments, and cytoplasmic proteins were obtained. Fig. 7d reports the western blot analysis of the lysates obtained from treated and untreated cells (see the Materials and methods). As shown in Fig. 7d, the intensity of the signal due to the Ubq antibody is significantly lower in the lysate from cells treated with 0.2 mg mL^−1^ NPs as compared to untreated cells (Fig. S4†). In particular, the analysis of the band intensity with image lab software reveals that the ubiquitination reaction in 0.2 mg mL^−1^ treated cells is 40% lower as compared to untreated cells (Fig. 7e).

Overall, the here reported cell data complement the *in vitro* results in demonstrating the capability of NPs to enter eukaryotic cells and to influence in a significant manner one of the key metabolic processes at the base of cell viability.

## Conclusions

Nanoparticles have gained a lot of attention from the scientific community, especially in the field of delivery systems for *in vivo* applications.^45^ Their use potentially allows the transport of the incorporated substances to defined body areas, thus facilitating targeted therapies with a low risk of side effects due to highly active or toxic substances. Such attention in the scientific community has been recently further amplified by the risks to human health associated with the use of nanoparticles in medical applications and by a phenomenon that in the beginning had consideration only in terms of environmental pollution:^2^ humans, like animals, are continuously exposed to the nanoparticles released by plastic debris (NPs), spread through the biosphere.^46,47^

It is now commonly established that NPs interact with biomolecules but the effects of these interactions remain poorly explored.

Proteins associate with NP generating the so-called protein corona that shields its surface and mainly define the biological identity of the particle^37^ leading to the *in vivo* response.^48^ It is thus highly desirable to understand the NP behavior when in the presence of proteins,^36^ and the effects of protein mediated transformation of NPs and their toxicity using controlled reference systems. For these reasons, we have here investigated by means of CD, TEM and NMR the *in vitro* interaction between polystyrene NPs and human ubiquitin. Our findings demonstrate that NPs and Ubq greatly influence each other upon interaction. In particular, we demonstrate that Ubq interacts with NPs using its C-terminal tail and the last three β-strands of its five-stranded β-sheet. This interaction induces the formation of a corona that uniformly surrounds the NPs. Interestingly, in line with studies reporting the interaction of Ubq with different nanoparticles,^16,32,38^ the Ubq undergoes a global reorganization of its structure,^33^ which allows the formation of protein–protein interactions^34^ that bridge different Ubq monomers to thicken the corona and induce NP coalescence of three or more coronated-NPs to produce larger particles. The interaction of NPs with human serum albumin was also shown to lead to the same phenomena: protein corona formation, protein-induced coalescence of NPs and structural changes in the protein.^11,14,34^

The C-terminal part of ubiquitin is responsible of the E1–Ubq conjugation, the crucial step of the ubiquitination process that we demonstrate to be influenced by the NP interaction with cells. Ubiquitination mis-function is at the bases of many neurodegenerative and oncogenic diseases;^49–51^ therefore, these results indicate that the exposure of cells to polystyrene nanoparticles, affecting cell ubiquitination processes, may represent a serious harm to the human health.

Our analysis offers an atomic detail and a mechanistic insight into the NP–Ubq interaction and demonstrates the potential of an approach that integrates different resolution techniques (spectroscopic techniques with TEM data) *in vitro* for predicting *in vivo* behavior.

The ability of NPs to induce protein conformational changes in the corona could be one of the mechanisms of NP nanotoxicity. As it is quite plausible that more proteins may be affected in a similar way, our present study represents a new framework in which such interactions can be characterized.

Since the protein corona and NPs along with the transformation and toxicity of NPs are still poorly understood, future studies are clearly needed for assessing the effects on human health of bio-accumulation and bio-magnification of NPs.

## Author contributions

MEE and RF conceived the study. GM, CI, LR, RA, MEE, MC and RF designed CD and NMR experiments. MdV and GDA performed CD and NMR experiments and analyzed the data. GG and RC conducted and analyzed TEM experiments. MTG and SDG performed and analyzed biochemical assays. All authors discussed the experimental results and analyses. MdV, GM, MEE and RF wrote the manuscript. All authors read and approved the final manuscript.

## Conflicts of interest

There are no conflicts to declare.

## Supplementary Material

SC-013-D2SC04434J-s001
