# Supplementary material for: Polystyrene nanoplastics affect the human ubiquitin structure and ubiquitination in cells: a high-resolution study
Source: Chem Sci. 2022 Nov 11;13(45):13563–73. doi: 10.1039/d2sc04434j (PMC9682910; doi:10.1039/d2sc04434j)
Supplement: SC-013-D2SC04434J-s001 [file SC-013-D2SC04434J-s001.pdf]

Electronic Supplementary Material (ESI) for Chemical Science.
This journal is © The Royal Society of Chemistry 2022

Polystyrene nanoplastics affect human ubiquitin structure and ubiquitination in cell: a high-resolution study

M. della Valle[1], G. D'Abrosca[1], M. T. Gentile[1], L. Russo[1], C. Isernia[1], S. Di Gaetano[2], R. Avolio[3], R. Castaldo[3], M. Cocca[3], G. Gentile[3], G. Malgieri[1], M. E. Errico[3] and R. Fattorusso[1]**

[1]Department of Environmental, Biological and Pharmaceutical Science and Technology, University of Campania - Luigi Vanvitelli, via Vivaldi 43, 81100 Caserta, Italy
[2] Institute of Biostructures and Bioimaging-CNR, Via Mezzocannone 16, 80134 Naples, Italy
[3]Institute for Polymers, Composites and Biomaterials - CNR, via Campi Flegrei, 34, 80078 Pozzuoli, Naples, Italy

* correspondence to: roberto.fattorusso@unicampania.it, mariaemanuela.errico@ipcb.cnr.it

Supplementary Material

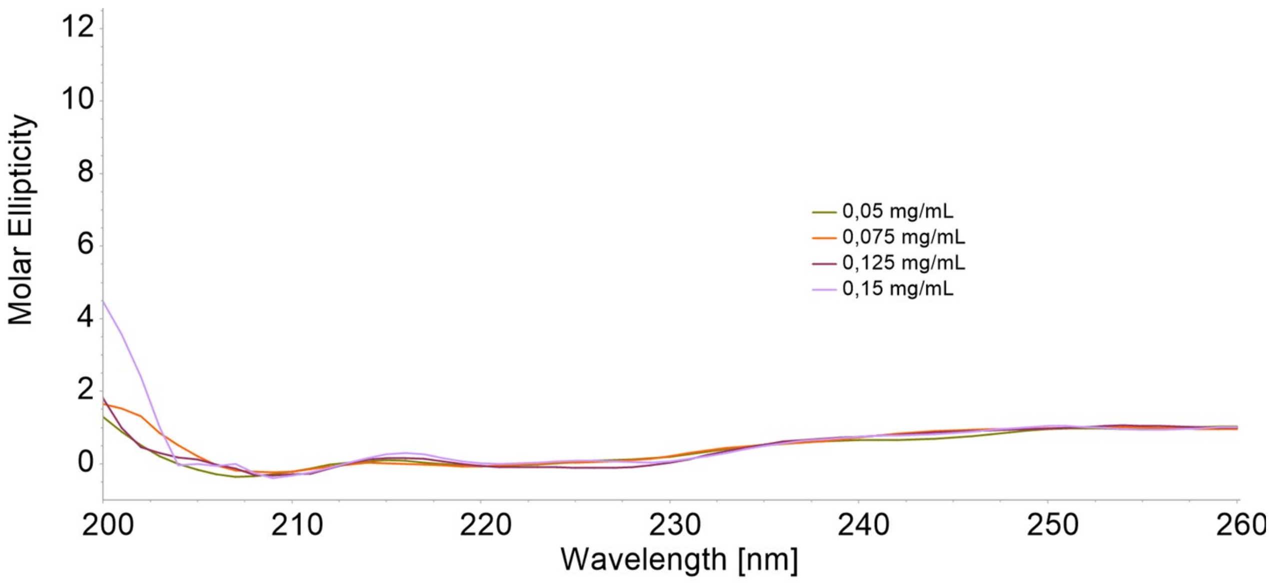

Figure S1: Far-UV CD spectra of PS-NPs at different concentrations.

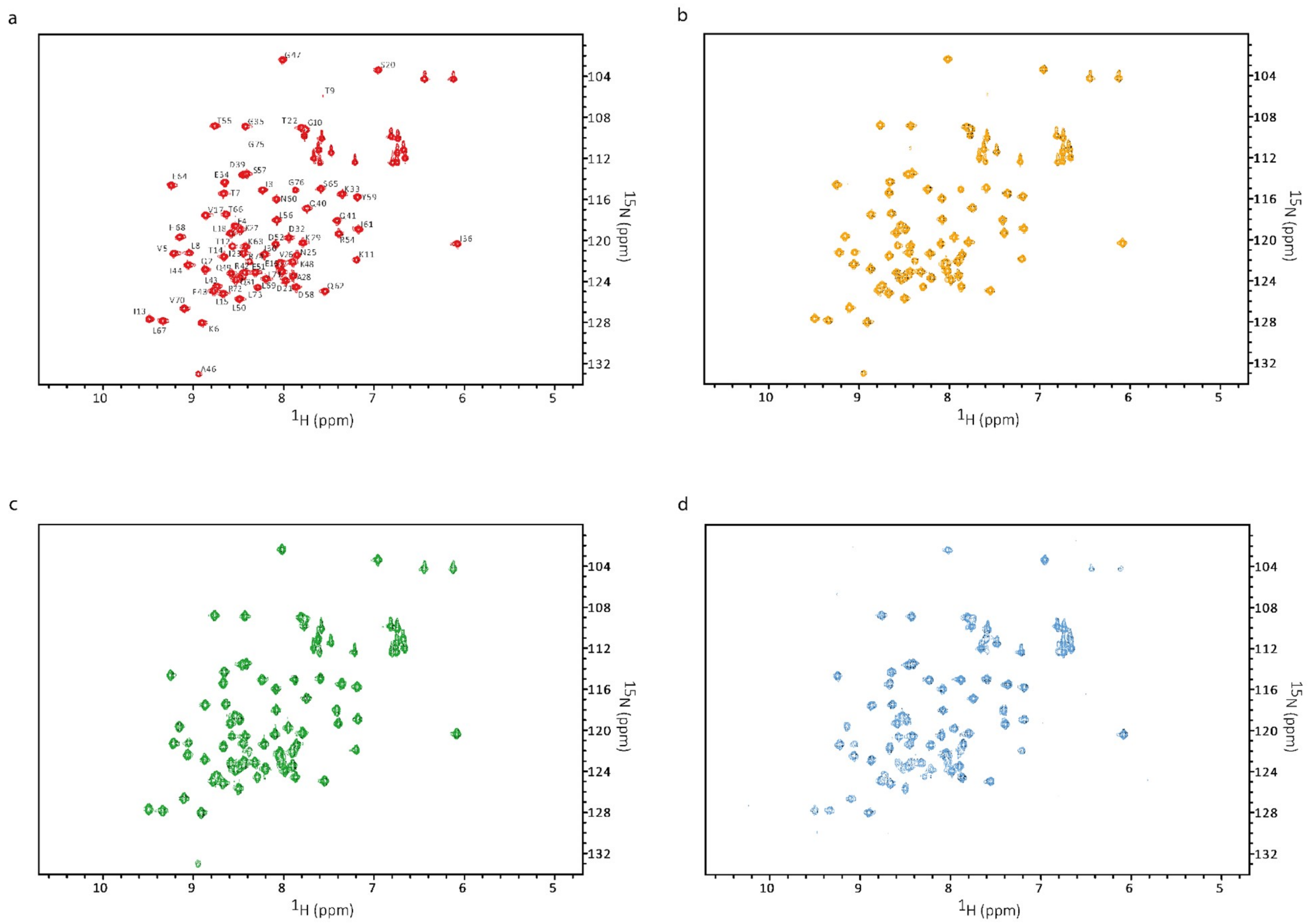

Figure S2: ^{1}H-^{15}N HSQC spectra of Ubq free (a) and in presence of 0,1 (b), 0,2 (c) and 0,3 (d) mg/mL of PS-NPs. Panel (a) reports the protein assignment.

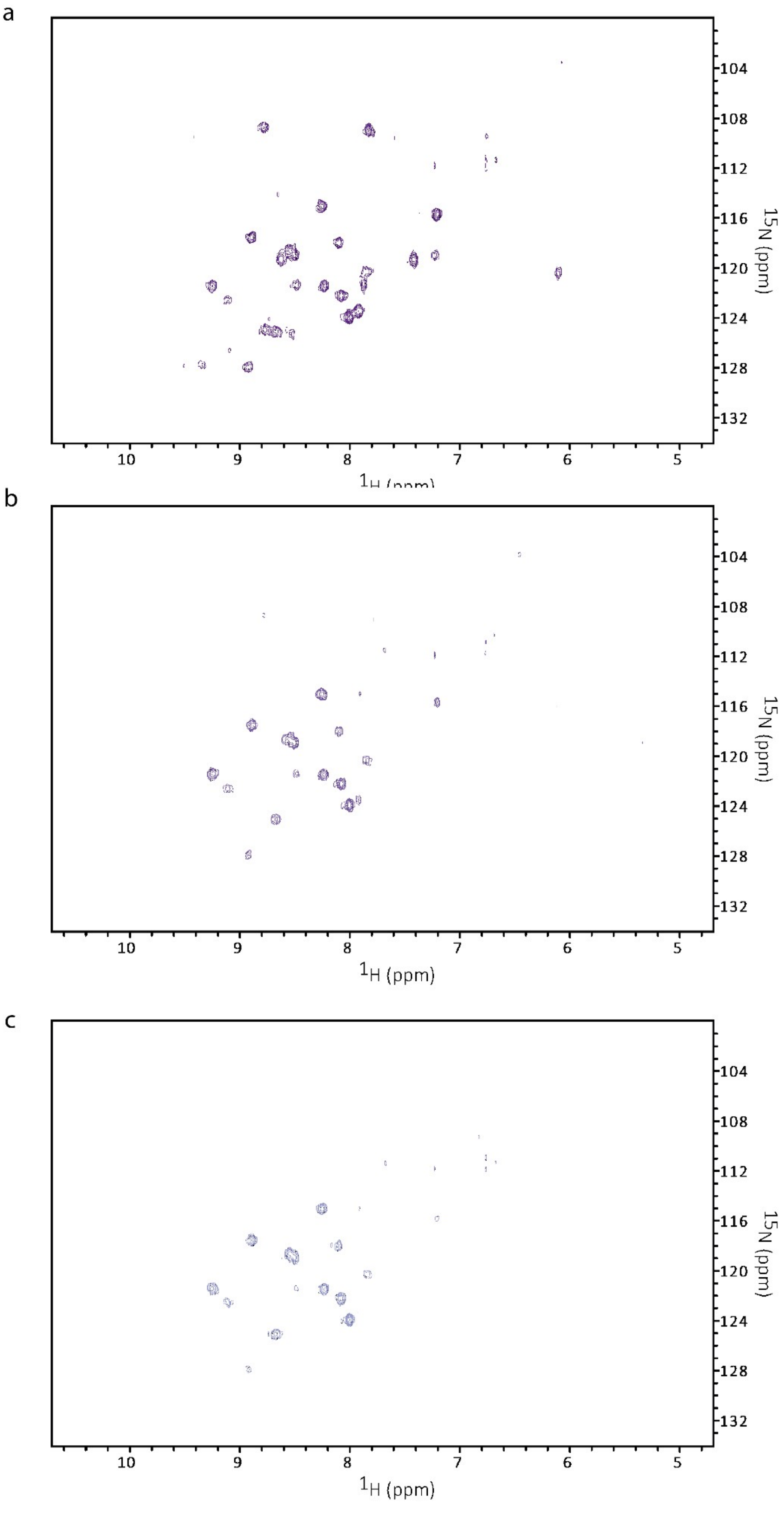

Figure S3: H-DX experiment. ^{1}H-^{15}N HSQC spectra of Ubq in presence of PS-NPs acquired after (a) 20 minutes, (b) 220 minutes and (c) 360 minutes.

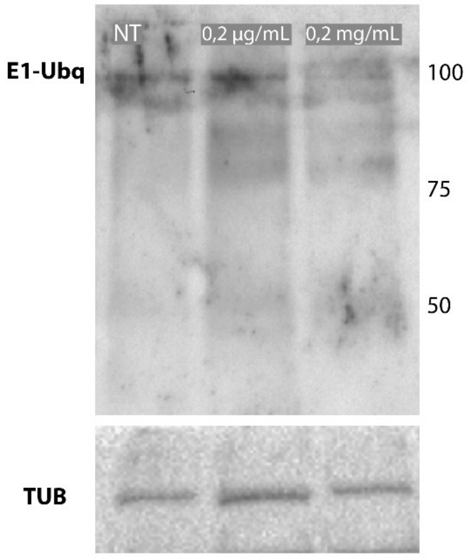

Figure S4: Western blotting analysis replica of Ubq signal in HeLa cells untreated (NT) and treated with increasing doses of PS-NPs (0,2 µg/mL and 0,2 mg/mL) for 24 hours.
